# Anemia in Ugandan pregnant women: a cross-sectional, systematic review and meta-analysis study

**DOI:** 10.1186/s41182-021-00309-z

**Published:** 2021-03-01

**Authors:** Felix Bongomin, Ronald Olum, Andrew Peter Kyazze, Sandra Ninsiima, Gloria Nattabi, Lourita Nakyagaba, Winnie Nabakka, Rebecca Kukunda, Phillip Ssekamatte, Davis Kibirige, Stephen Cose, Annettee Nakimuli, Joseph Baruch Baluku, Irene Andia-Biraro

**Affiliations:** 1 Department of Medicine, School of Medicine, Makerere University College of Health Sciences, Kampala, Uganda; 2 Department of Medical Microbiology & Immunology, Faculty of Medicine, Gulu University, Gulu, Uganda; 3 School of Medicine, Makerere University College of Health Sciences, Kampala, Uganda; 4 Department of Medicine, Uganda Martyrs Hospital Lubaga, Kampala, Uganda; 5 Department of Immunology and Molecular Biology, School of Biomedical Sciences, Makerere University College of Health Sciences, Kampala, Uganda; 6 Medical Research Council/Uganda Virus Research Institute and London School of Hygiene and Tropical Medicine Uganda Research Unit, Entebbe, Uganda; 7 Directorate of Programs, Mildmay Uganda, Wakiso, Uganda; 8 Department of Obstetrics & Gynecology, School of Medicine, Makerere University College of Health Sciences, Kampala, Uganda; 9 Department of Internal Medicine, Mulago National Referral Hospital, Kampala, Uganda; 10 Department of Clinical Research, Faculty of Infectious and Tropical Disease (ITD), London School of Hygiene and Tropical Medicine, London, UK

**Keywords:** Anemia, Pregnancy, Uganda

## Abstract

**Background:**

Anemia in pregnancy represents a global public health concern due to wide ranging maternal and neonatal adverse outcomes in all peripartum periods. We estimated the prevalence and factors associated with anemia in pregnancy at a national obstetrics and gynecology referral hospital in Uganda and in addition performed a systematic review and meta-analysis of the overall burden of anemia in pregnancy in Uganda.

**Methods:**

We conducted a cross-sectional study among 263 pregnant women attending the antenatal care clinic of Kawempe National Referral Hospital, Kampala, Uganda, in September 2020. Anemia in pregnancy was defined as a hemoglobin level of < 11.0 g/dl and microcytosis as a mean corpuscular volume (MCV) of < 76 fL. We also performed a systematic review (PROSPERO Registration ID**:** CRD42020213001) and meta-analysis of studies indexed on MEDLINE, Embase, African Journal Online, ClinicalTrials.gov, ICTRP, and the Cochrane Library of systematic review between 1 January 2000 and 31 September 2020 reporting on the prevalence of anemia in pregnancy in Uganda.

**Results:**

The prevalence of anemia was 14.1% (*n*= 37) (95%CI 10.4–18.8), of whom 21 (56.8%) had microcytic anemia. All cases of anemia occurred in the second or third trimester of pregnancy and none were severe. However, women with anemia had significantly lower MCV (75.1 vs. 80.2 fL, *p*<0.0001) and anthropometric measurements, such as weight (63.3 vs. 68.9kg; *p*=0.008), body mass index (25.2 vs. 27.3, p=0.013), hip (98.5 vs. 103.8 cm, *p*=0.002), and waist (91.1 vs. 95.1 cm, *p*=0.027) circumferences and mean systolic blood pressure (BP) (118 vs 125 mmHg, *p*=0.014). Additionally, most had BP within the normal range (59.5% vs. 34.1%, *p*=0.023). The comparison meta-analysis of pooled data from 17 published studies of anemia in pregnancy in Uganda, which had a total of 14,410 pregnant mothers, revealed a prevalence of 30% (95% CI 23–37).

**Conclusions:**

Despite our study having a lower prevalence compared to other studies in Uganda, these findings further confirm that anemia in pregnancy is still of public health significance and is likely to have nutritional causes, requiring targeted interventions. A larger study would be necessary to demonstrate potential use of basic clinical parameters such as weight or blood pressure as screening predictors for anemia in pregnancy.

**Supplementary Information:**

The online version contains supplementary material available at 10.1186/s41182-021-00309-z.

## Introduction

Anemia in pregnancy, defined as hemoglobin level less than 11 g/dl, is a serious public health problem that is estimated by the World Health Organization (WHO) to affect approximately 40% of pregnant women globally [1]. From the global burden of disease study, there has been a slight decrease in the prevalence of anemia from 43% (39–47%) to 38% (34–43%) among pregnant women compared to 33% (29–38%) to 29% (24–35%) in non-pregnant women between 1995 and 2011 [2]. This prevalence translates to about 32 million (28 to 36 million) pregnant women with anemia globally [2].

In developing countries like Uganda, mortality during pregnancy (maternal and neonatal) is still high despite a steady decline in recent years. The maternal mortality ratio in Uganda is as high as 336 deaths per 100,000 live births according to the Uganda Demographic Health Survey (UDHS), 2016 [3]. Neonatal mortality is defined as “the probability of dying within the first month of life is 27 deaths per 1000 live births and the mortality rate under five years is at 64 deaths per 1000 live births” [3]. The etiology of anemia in pregnancy is multifactorial and may result from physiological changes in which plasma volume increases relative to red cell mass expansion resulting in hemodilution, or from acquired and inheritable disorders that may occur prior or during pregnancy [4–7]. Irrespective of the etiology, anemia in pregnancy has been associated with serious adverse pregnancy outcomes, including high maternal and perinatal morbidity and mortality, antenatal and postnatal sepsis, maternal transfusion requirement, impaired cognitive development in children, increased risk of small for gestation age and low birth weight neonates, and prematurity [8, 9]. Moreover, anemia is associated with all-cause mortality even in the absence of comorbid conditions [10].

A few studies have attempted to establish the prevalence, associated factors, and consequences of anemia in pregnancy in Uganda [11–14]. From a previous systematic review, the burden of anemia in pregnancy in East African region was estimated at 36% [2]. However, in the past 5 years, there has been a significant improvement in antenatal care services across the region and in Uganda in particular. Consequently, the prevalence of anemia is expected to have declined over the years.

In the present study, we aimed to determine the prevalence of anemia among pregnant women at Kawempe National Referral Hospital (KNRH) and to conduct a systematic review and meta-analysis to precisely define the prevalence of anemia in Uganda as a whole. The findings are vital in the design of policies and strategies aimed at reducing the burden of anemia in pregnancy, and hence lowering the maternal mortality ratio in Ugandan context.

## Methods

### Study design

We conducted a single-center cross-sectional study and a systematic review and meta-analysis to determine the prevalence of anemia among pregnant women in Uganda.

### Cross-sectional study

An antenatal care-based cross-sectional study was conducted at KNRH, a large specialized obstetrics and gynecology referral hospital in Kampala, Uganda, in September 2020. KNRH is located 8 km from Kampala’s central business district, along the Kampala-Gulu Highway, and has 170 beds. Besides serving the population within its location, it is also a national referral hospital receiving referrals mainly from lower health centers in Kampala, Wakiso, and neighboring districts. All mothers receive a standard antenatal care package, including prenatal ultrasonography, and high-risk mothers are offered special investigations such as genetic testing or biophysical profiling as indicated. The antenatal care clinic at KNRH runs on Tuesday through Thursday every week, offering antenatal care services to about 50–60 new mothers every clinic day. We enrolled pregnant women who were willing and competent to provide informed written consent, regardless of gestational age or gravidity. Women living with sickle cell anemia were excluded. Trained study nurses consecutively enrolled eligible participants until the sample size was reached.

#### Study measurements

A study assistant administered a semi-structured study questionnaire (Supplementary File 1) through a face-to-face interview to collect information on maternal characteristics such as age, gravidity, education level, occupation, marital status, HIV status, tuberculosis contact, gestational age, history of abortion, smoking and alcohol usage and the number of antenatal care visits in the current pregnancy. Study variables to include in the questionnaire were guided by previous studies in Uganda [11, 12]. Gestation was estimated using the date of the last normal menstrual period. A study nurse drew 4ml of blood and samples were analyzed using HumaCount 5D Hematology System (Wiesbaden, Germany) at Paramount Hospital Kampala laboratory. Anemia in pregnancy was defined using the WHO classification as Hb <11 g/dl and further classified into mild (Hb 10.0–10.9 g/dL), moderate (Hb 7.0–9.9g/dL), and severe (Hb <7.0g/dL) [1].

Body mass index (weight [kg]/ (height [m])^2^) and waist-hip ratio (waist circumference (cm)/hip circumference (cm)) were calculated following anthropometric measurements. Specifically, weight was measured with minimal clothing and without shoes using a digital bathroom weighing scale (SECA-Germany) while height measured used a stadiometer (Fazzini S208 height rod). The waist and hip circumferences were measured using a tailor’s measuring tape. The brachial blood pressure (BP) was measured on both arms using MEDQUIP® arm-type fully automatic digital blood pressure monitor (Model: BP-2400) with an appropriate adult cuff size and the participant seated upright in a comfortable position. The average of the two measurements was considered as the participant’s blood pressure. BP was classified according to the 8th Joint National Committee (JNC-8), as normal (BP = <120/<80 mm Hg), elevated (BP = 120–129/<80 mm Hg), stage 1 hypertension (BP= 130–139 or 80–89 mm Hg), and stage 2 hypertension (BP= ≥140 or ≥90 mm Hg) [15].

#### Sample size estimation and statistical analysis

Using Kish-Leslie’s formula (Kish-Leslie) [16], we calculated a sample size of 263 participants based on an estimated prevalence of anemia in pregnancy at 22% in 2 Ugandan regional referral hospitals [11], a margin of error of 5%, and a *z*-statistics at 95% confidence interval (95% CI).We applied the Shapiro-Wilk normality test to evaluate all quantitative variables to select the appropriate test. Categorical variables were expressed as frequencies and percentages. Parametric data were summarized as mean and standard deviations (mean ± SD) and non-parametric data as median and range. Chi-squared or Fischer’s exact tests were used to assess for associations between anemia and categorical variables while Mann-Whitney *U*/Student’s *t* tests and Wilcoxon-signed rank/analysis of variance (ANOVA) were used to assess for associations between anemia and continuous variables (age, blood pressure, weight, age, gestational age, height, waist and hip circumferences). All variables with *p*<0.2 in the bivariate analyses were fitted into a multivariate logistic regression model to adjust for potential confounders such as age, parity, gestational age, and HIV status. Multivariable logistic regression model was used to assess for independent predictors of anemia in pregnancy.

### Systematic review

#### Search strategy and study eligibility criteria

We performed a systematic review and meta-analysis in accordance to the Preferred Reporting Items for Systematic Review and Meta-Analysis (PRISMA) checklists [17]. The PRISMA checklist is provided as Supplementary File 2. This study was prospectively registered on the PROSPERO database (*Registration ID:* CRD42020213001).

The search was conducted by systemically identifying articles published from 1 January 2000 to 31 September 2020. The year 2000 was chosen as the start for the period under evaluation because around this time the Uganda Ministry of Health developed their first national anemia policy [18]. Eligible studies were observational studies reporting on the prevalence of anemia among pregnant women of all gestational ages in Uganda within the study period above. We excluded case reports and case series, reviews and meta-analyses, animal studies, and protocols. We also excluded papers that reported anemia among women in the pre-natal and post-natal periods.

We explored Embase, MEDLINE (through PubMed), African Journals Online (AJOL), and the Cochrane Library of systematic review for eligible studies published in English. The Medical Subject Headline terms searched are provided as a Supplementary File 3. Authors of eligible articles where only abstracts were provided were contacted. Also, we performed a manual search of references on all citations that met the inclusion criteria for our study.

The search outputs were run through Healthcare Databases Advanced Search (National Institute for Health and Care Excellence, UK) program in order to remove duplicate research articles. Screening of titles and abstracts to isolate eligible studies were done by FB and RO. Thereafter, we retrieved and discussed the full texts of potentially eligible papers. Any disagreements about eligibility of the articles for the study were resolved by consensus among the authors.

Two independent reviewers extracted data (JBB and APK), which was subsequently coded. We used a data extraction form prepared using Microsoft Excel 2016 to collect information from all eligible studies such as year of publication, first author’s name, sample size, method of recruitment, study design, prevalence, etiology, and risk factors of anemia. When the required data was not readily available from published articles, we requested raw data from the authors. Two other independent reviewers reviewed the extracted data, and any disagreements were resolved by consensus (FB and RO). The risk of bias of individual studies was assessed by three reviewers (SN, FB, and RO) using the modified Newcastle-Ottawa Scale for cross-sectional studies with a maximum of 10 stars for each study. All studies included in the systematic review and meta-analysis scored 7 stars and above indicating good quality (Supplementary File 4).

#### Data synthesis and statistical analysis

We used both a qualitative and quantitative synthesis to present the key findings of the selected studies. In the quantitative synthesis, a random-effect model meta-analysis was performed using *meta* command for analysis of proportions and presented as prevalence, 95% CIs, and weights. Results of the meta-analysis were presented in forest plots. Heterogeneity across studies was assessed using *Q* statistics and results presented as *I*^2^ indices and *p* values. Publication bias was assessed using a funnel plot and sensitivity analyses performed.

For both methodological approaches, STATA version 16 (StataCorp LLC) was used for data analysis, all analyses were two-tailed, and *P*<0.05 was considered significant at a 95% CI.

## Results

### Cross-sectional study

#### Socio-demographic characteristics

Out of the 263 eligible participants, the median (range) age was 26 (16–40) years, and 156 (59.3%) women were aged ≥ 25 years. The majority (64.6%, *n*=174) were attending antenatal care for the first time. Most (90.1%, *n*=237) of the pregnant women were married and had at least secondary education (77.9%, *n*=205). With regard to occupation, 99 (37.6%) were unemployed/housewives. Seven (2.7%) participants had HIV infection. One hundred and sixty-eight (63.9%) pregnant women were multiparous. Only 12 (4.6%) respondents were in their 1st trimester (Table 1).

**Table 1:** Bivariate analysis for socio-demographic and anthropometric factors associated with anemia in pregnancy

Participant variable	ALL (*n*=263)	Normal Hb (*n*=226)	Anemia (*n*=37)	*p* value
	Number of cases (%)/median (range)	Number of cases (%)/mean ± SD	Number of cases (%)/mean ± SD	
Antenatal care visit at enrollment				
First	170 (64.6)	143 (63.3)	27 (73)	0.307
Second	31 (11.8)	27 (11.9)	4 (10.8)	
Third	19 (7.2)	19 (8.4)	0 (0)	
Fourth and more	43 (16.4)	37 (16.4)	6 (16.2)	
Age				
< 25 years	107 (40.7)	88 (38.9)	19 (51.4)	0.154
≥ 25 years	156 (59.3)	138 (61.1)	18 (48.6)	
Marital status				
Married	237 (90.1)	205 (90.7)	32 (86.5)	0.725
Single	16 (6.1)	13 (5.8)	3 (8.1)	
Widowed	10 (3.8)	8 (3.5)	2 (5.4)	
Education level				
Informal	4 (1.5)	4 (1.8)	0 (0)	0.612
Primary	54 (20.5)	47 (20.8)	7 (18.9)	
Secondary	141 (53.6)	118 (52.2)	23 (62.2)	
Tertiary	64 (24.3)	57 (25.2)	7 (18.9)	
Occupational status				
Business	113 (43)	99 (43.8)	14 (37.8)	0.132
Professional	51 (19.4)	47 (20.8)	4 (10.8)	
Unemployed	99 (37.6)	80 (35.4)	19 (51.4)	
Smoking status				
Former	3 (1.1)	2 (0.9)	1 (2.7)	0.367
Never	260 (98.9)	224 (99.1)	36 (97.3)	
Alcohol usage				
Current	12 (4.6)	12 (5.3)	0 (0)	0.348
Former	44 (16.7)	38 (16.8)	6 (16.2)	
Never	207 (78.7)	176 (77.9)	31 (83.8)	
District of residence				
Kampala	184 (70)	158 (69.9)	26 (70.3)	0.214
Wakiso	72 (27.4)	62 (27.4)	10 (27)	
Mukono	4 (1.5)	4 (1.8)	0 (0)	
Entebbe	1 (0.4)	1 (0.4)	0 (0)	
Luweero	1 (0.4)	0 (0)	1 (2.7)	
Mpigi	1 (0.4)	1 (0.4)	0 (0)	
Residence				
Urban	184 (70)	162 (71.7)	22 (59.5)	0.133
Rural	79 (30)	64 (28.3)	15 (40.5)	
HIV status				
Negative	256 (97.3)	220 (97.3)	36 (97.3)	1.000
Positive	7 (2.7)	6 (2.7)	1 (2.7)	
Family size				
≤4	210 (79.9)	179 (79.2)	31 (83.8)	0.520
≥5	53 (20.2)	47 (20.8)	6 (16.2)	
Gravidity				
Primigravida	95 (36.1)	80 (35.4)	15 (40.5)	0.635
Multigravida	138 (52.5)	120 (53.1)	18 (48.6)	
Grand multigravida	23 (8.8)	19 (8.4)	4 (10.8)	
Great grand multigravida	7 (2.7)	7 (3.1)	0 (0)	
Previous abortion				
No	223 (84.8)	190 (84.1)	33 (89.2)	0.621
Yes	40 (15.2)	36 (15.9)	4 (10.8)	
Gestation age at enrollment (weeks)	28 (5–40)	26.7 ± 7.3	7.3 ± 5.3	0.347
Trimester at enrollment				
1	12 (4.6)	12 (5.3)	0 (0)	0.200
2	115 (43.7)	101 (44.7)	14 (37.8)	
3	136 (51.7)	113 (50)	23 (62.2)	
Anthropometry				
Weight (kilograms)	65.5 (41.9–117.6)	68.9 ± 12.4	63.3 ± 8.6	0.008*
Height (meters)	160 (140–175)	159.1 ± 6.3	158.6 ± 5.9	0.658
BMI (kg/m^2^)	26.5 (17.8–43.2)	27.3 ± 4.7	25.2 ± 3.6	0.013*
Waist circumference (centimeters)	95 (60–138)	95.1 ± 10.4	91.1 ± 8.7	0.027*
Hip circumference (centimeters)	102 (66–136)	103.8 ± 9.8	98.5 ± 8.0	0.002*
WHR	0.92 (0.59–1.38)	0.9 ± 0.1	0.9 ± 0.1	0.576
Blood pressure at enrollment				
SBP (mmHg), average	124 (93–267)	125 ± 18	118 ± 10	0.014*
DBP (mmHg), average	77 (57–180)	77 ± 13	74 ± 8	0.070
Blood pressure category				
Normal	99 (37.6)	77 (34.1)	22 (59.5)	0.023*
Elevated	54 (20.5)	48 (21.2)	6 (16.2)	
Hypertension I	79 (30.0)	70 (31)	9 (24.3)	
Hypertension II	28 (10.7)	28 (12.4)	0 (0)	
Hypertensive crisis	3 (1.1)	3 (1.3)	0 (0)	

*Statistically significant at *P*<0.05

##### Prevalence of anemia

The prevalence of anemia was 37/263 (14.1%; 95% CI 10.4–18.8). Of these, 25 (67.6%) were classified as mild, 12 (32.4%) moderate, and none of the women had severe anemia. The median Hb was 10.3g/dL (range 7.8–10.9). The prevalence of anemia was highest (62.2%) among women in their 3rd trimester followed by those in the 2nd trimester (37.8%); none of the mothers had anemia in the 1st trimester. The majority (56.8%, *n*=21) of those with anemia had microcytosis (mean corpuscular volume [MVC] < 76 fL). The mean MCV for women with anemia was significantly lower than for those without (75.1 vs. 80.2 fL, *p*<0.0001).

#### Association between anemia and maternal socio-demographic and anthropometric variables

Compared to pregnant women with normal hemoglobin levels, women with anemia had lower anthropometric measurements: weight (63.3 kg vs 68.9 kg; *p*=0.008), lower BMI (25.2 vs. 27.3, *p*=0.013), smaller hip (98.5 cm vs. 103.8 cm, *p*=0.002), and waist (91.1 cm vs. 95.1 cm, *p*=0.027) circumferences. Additionally, they had lower mean systolic BP (118 mmHg vs 125 mmHg, p=0.014), and most of them had normal overall BP (59.5% vs 34.1%, *p*=0.023) as shown in Table 1. In multivariable logistic regression analysis, none of the variables showed a statistically significant association after adjusting for confounders.

### Systematic review and meta-analysis

#### Characteristics of eligible studies

**Fig. 1:**
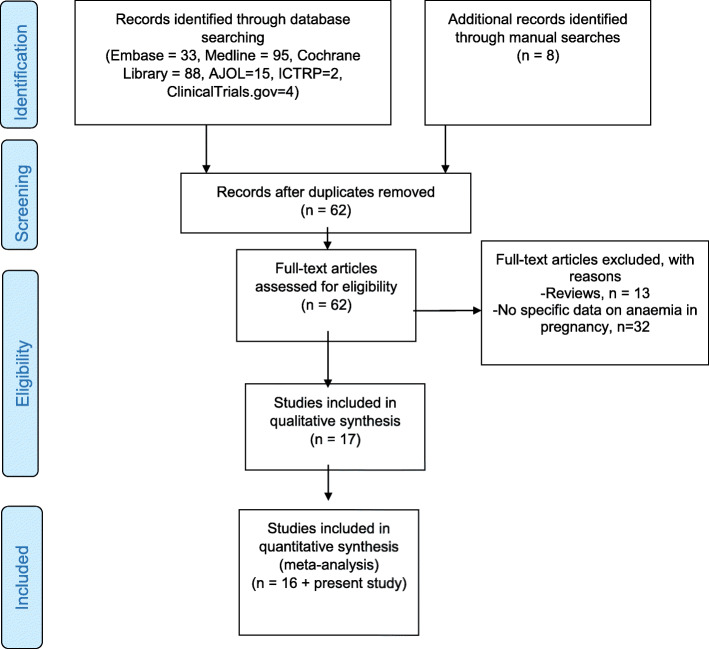
PRISMA flow diagram

A total of 18 studies, including the present study, were included in the qualitative synthesis and 17 studies were eligible for meta-analysis (Fig. 1). The majority of these studies (10/18, 55.6%) were conducted in central Uganda. Table 2 summarizes the characteristics of studies included in the review and meta-analysis. Overall, the studies included 14,410 patients.

**Table 2:** Characteristics of studies included in the systematic review and meta-analysis

Study ID	Study type	District (region)	Population	Study period	Total	Cases	Prevalence	Malaria	HIV	Helminths
Bongomin (present study)	Cross-sectional	Kampala (Central)	Antenatal care	2020	263	37	14.1%	NA	7	NA
Finkelstein, 2020 [14]	Prospective sub study in an RCT	Tororo (Eastern)	Pregnant women living with HIV	2009 to 2013	367	114	31.1%	NA	All were HIV+	NA
Mahamoud, 2020 [19]	Cross-sectional	Kampala (Central)	Antenatal care	2016	345	89	25.8%	NA	NA	NA
Nekaka, 2020 [20]	Cross-sectional	Mbale (Eastern)	The women who attended antenatal care and for labor at term	2017 to 2018	210	61	29.0%	(9.1%) tested positive for malaria RDT		
Okia, 2019 [21]	Cross-sectional	Ntungamo (Western)	Antenatal care	2018	163	12	7.4%	NA	NA	NA
Obai, 2016 [11]	Cross-sectional	Gulu and Hoima (North and West)	Antenatal care	2012	743	164	22.1%	NA	NA	NA
Braun, 2015 [22]	Cross-sectional	Fort Portal (Western)	Antenatal care	2013	692	200	28.9%	30/63 had malaria vs 170/629 without malaria	Not documented	NA
Baingana, 2014 [23]	Cross-sectional	Kampala (Central)	HIV-negative women in their first or second attending antenatal care.	2009	141	41	29.1%	6/8 with malaria had anemia vs 38/143 without malaria	Negative	5/8 with hookworm had anemia vs 38/139 without hookworms
Ononge, 2014 [12]	Cross-sectional	Mpigi (Central)	Pregnant women at 28+ weeks of gestation at six health facilities.	2013	2436	791	32.5%	Anemia in pregnancy was significantly associated with malaria	190/2436 were HIV+; significant association with anemia.	NA
Arinaitwe, 2013 [24]	Cross-sectional	Tororo (Eastern)	Women delivering at Tororo District Hospital, with history of fansidar use.	2011	565	247	43.7%	19.1% of all participants had malaria	NA	NA
Mbule, 2013 [25]	Cross-sectional	Kiboga (Western)	Pregnant women in randomly selected household	Not indicated	304	191	62.8%	NA	NA	NA
Namusoke, 2010 [26]	Cross-sectional	Kampala (Central)	Pregnant women in labor	2004 to 2005	389	86	22.1%	Peripheral smear—9% (35/391), placental smear—11.3% (44/389), and placental histology- 13.9% (53/382)	NA	NA
Ndibazza, 2010 [27]	RCT	Entebbe (Central)	Pregnant women being recruited to a clinical trial	2003 to 2005	2507	994	39.6%	268/2507 (11%) had malaria at enrolment	299/2507 (12%) overall sample were HIV+ at enrolment	At enrollment, 68% of women had helminths, 45% had hookworm, 18% had *Schistosoma mansoni* infection
Mbonye, 2008 [28]	Trial	Mukono (Central)	Community-based study	Not indicated	761	431	56.6%	573/2344 had malaria at recruitment	NA	NA
Ndyomugyenyi, 2008 [29]	RCT	Masindi (Western)	Pregnant women of any parity attending antenatal care in their second trimester	2003 to 2005	832	171	20.6%	NA	NA	Majority infected with hookworm, Ascaris lumbricoides and Trichuris trichiura.
Muhangi, 2007 [13]	Sub study in a trial	Entebbe (Central)	Healthy pregnant women at enrolment to a trial of deworming in pregnancy.	2003 to 2005	3155	1277	40.5%	175/268 in malaria+ vs 807/2191 in malaria - patients, significant correlation	171/299 (57.2%) in HIV positive patients compared to 825/2208 in HIV - (37.2%), significant correlation	No significant correlations with hookworms and other parasites (*Strongyloides*, *Schistosoma*, etc.)
Kaye, 2006 [30]	Prospective cohort	Kampala (Central)	Pregnant women attending antenatal care in the second trimester and followed up to delivery.	2004 to 2005	612	433	70.8%	NA	NA	NA
Kasumba, 2000 [31]	Cross-sectional	Kampala (Central)	Pregnant women presenting at the labor ward for delivery	1998	537	39	7.3%	Overall prevalence of 8.6%		NA

*RCT* randomized clinical trial, *NA* not applicable, *HIV* human immunodeficiency virus

#### Pooled prevalence of anemia in pregnancy in Uganda

**Fig. 2:**
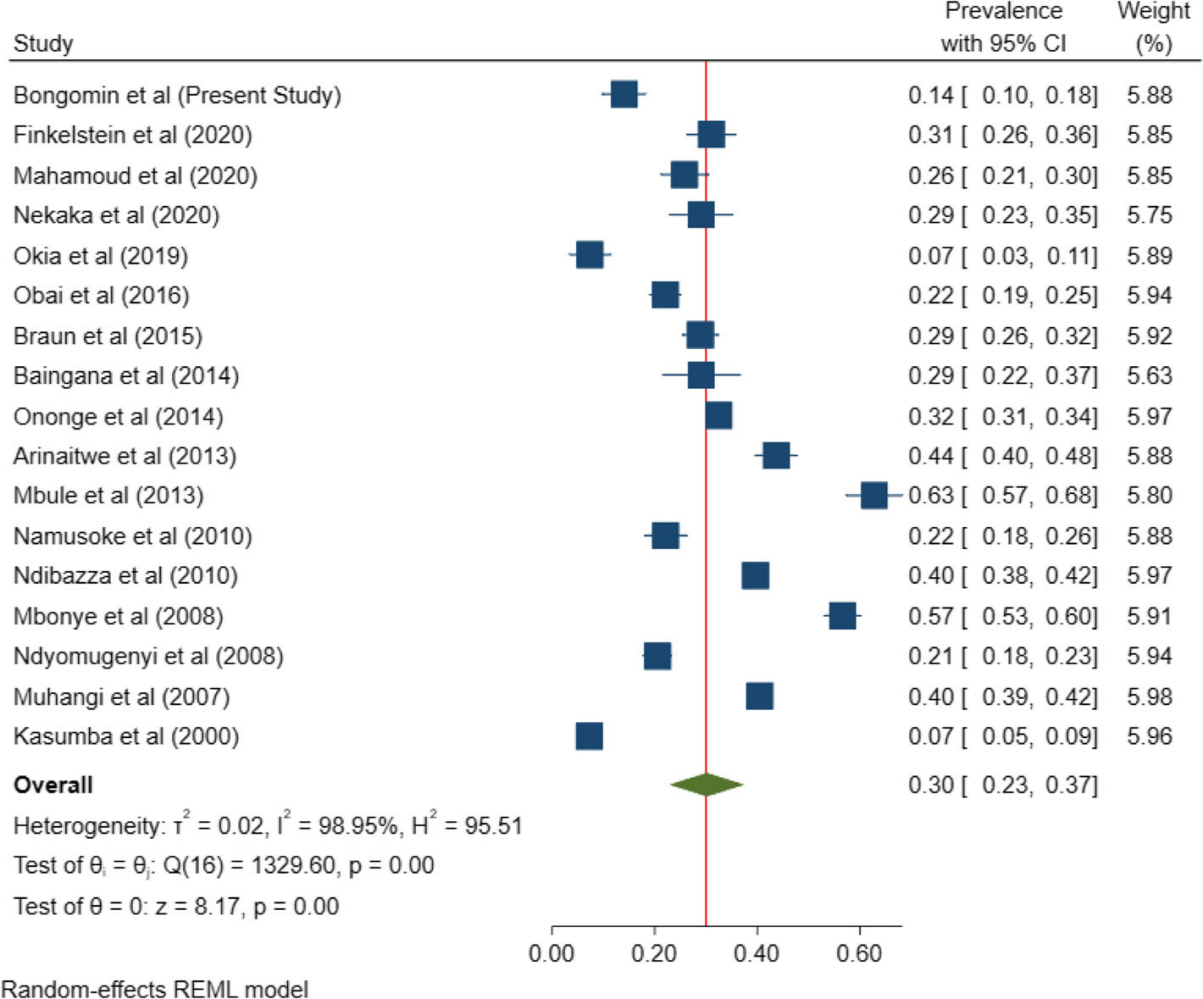
Pooled prevalence of anemia in pregnancy in Uganda

**Fig. 3:**
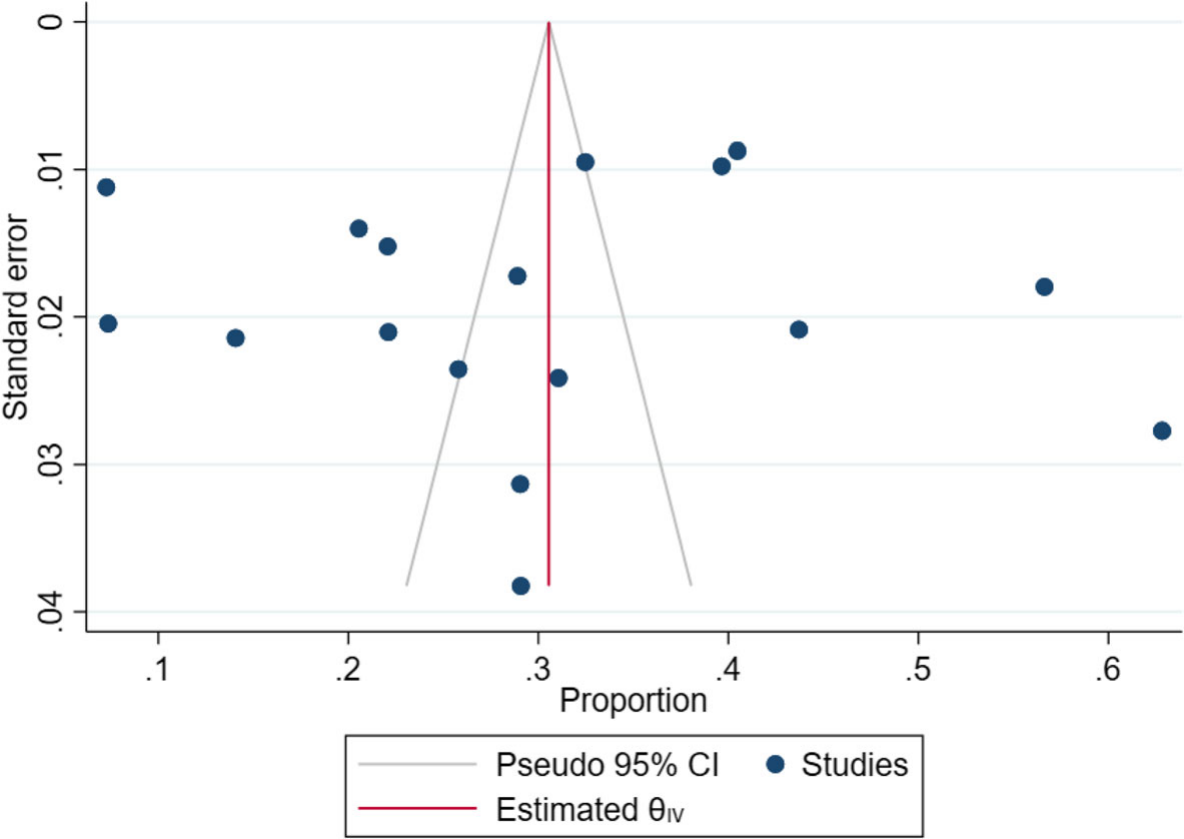
Funnel plot of studies reporting prevalence of anemia in pregnancy

**Fig. 4:**
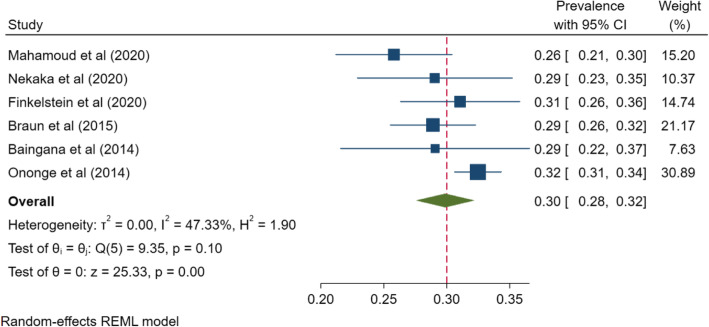
Sensitivity analysis of studies falling within the funnel plot

The overall, pooled prevalence of anemia among pregnant women in Uganda was 30% (95% CI 23–37%; *I*^2^=98.95%, *P*<.001) (Fig. 2). A funnel plot was generated to show the distribution of the included studies (Fig. 3). There was a very high degree of heterogeneity across studies. A sensitivity analysis of the 6 studies within the funnel yielded a pooled prevalence of 30% (95% CI 28–32%; *I*^2^= 47.33%, *p*<0.001) (Fig. 4). Anemia in pregnancy was related to malaria infection in 9 studies, HIV in 4 studies, and helminths infections in 4 studies.

## Discussion

Anemia is a common medical complication in pregnancy, affecting up to an estimated 40% of pregnant women compared to 29% of all women of reproductive age globally [1, 2]. Despite anemia being a global concern, women in less developed countries are disproportionately affected. As such, only about 5% of pregnant women in developed countries are estimated to be anemic compared to 80% in some developing countries [5, 32]. In the present cross-sectional study, we found a prevalence of anemia of 14.1% (95% CI 10.4–18.8), with no cases of severe anemia. However, the pooled prevalence of anemia in Uganda over a two-decade period was 30% (95% CI 23–37), a burden which is of moderate public health significance according to the WHO classification of public health significance of anemia [1]. In neighboring Kenya and Tanzania, the prevalence of anemia among pregnant women was estimated at 20.1 % [33] and 30.5% [34] respectively. Thus, there is a similar trend in the prevalence of anemia among pregnant women across the East African community.

From the Lancet’s global burden of diseases study, the prevalence of anemia among pregnant women in the East African region was estimated at 36% (95% CI 30–41) [2]. This is similar to the pooled prevalence observed in our meta-analysis but higher than the prevalence of anemia in our cross-sectional study. A similar study by Baingana et al. [23] from the same center as ours conducted in 2009 reported a prevalence of anemia of 29.1%. However, this study had a small size and the high prevalence may not have been a true reflection of the burden of gestational anemia at that time. Also, economic status has generally improved in Uganda coupled with improved nutrition and this may have resulted in an overall decline in the burden of nutritional deficiencies in Uganda [23]. A secondary analysis of data from three consecutive UDHS showed pooled prevalence of anemia among pregnant women of 43.2% in the period spanning 2006–2016 [35]. Our systematic review included studies that were predominantly conducted in central Uganda which has a relatively higher socio-economic status compared to the rest of the country [36]. This could explain why our pooled prevalence is lower than that reported from national demographic surveys.

Anemia in pregnancy is an important cause of maternal mortality and morbidity in Uganda [37]. Many risk factors have been associated with anemia in pregnancy, including age 15–24 years, family size of >5, multigravida, low socioeconomic status, current clinical illness, intestinal parasitic infection, being in the third trimester, history of menorrhagia, and low body mass index [32]. Other key risk factors include the high burden of helminthic, malaria, and HIV infections in Uganda [27, 38]. A recent study from Northern Uganda showed a high (11%) prevalence of hookworm infection among pregnant mothers at booking antenatal care attendance [39].

From our cross-sectional study, it was apparent that women with anemia predominantly had microcytic anemia. Additionally, they consistently had lower anthropometric measurements. Taken together, these findings suggest that iron deficiency arising from nutritional deficiency may be contributory to anemia in pregnancy although we did not perform iron studies or peripheral blood films. Although the Ministry of Health in Uganda recommends iron and folate supplementation during pregnancy, only 12% of women adhere to these medications [40]. Our findings highlight the need for screening for anemia and innovative ways to improve adherence to recommended iron supplementation. Additionally, clinicians need to consistently evaluate nutritional status of pregnant women and offer relevant nutritional advice. Besides anemia, it was evident from our study that over 60% of pregnant women had above normal blood pressure. The association between higher hemoglobin levels and elevated systolic blood pressure in pregnant women could be due to insufficient plasma expansion leading to disordered blood pressure hemodynamics [41]. While anemia in pregnancy is damaging, elevated maternal hemoglobin is also associated with gestational hypertensive disorders and poor birth outcomes [41]. Pregnancy-related hypertensive disorders are associated with long-term risk for cardiovascular events, renal disease, diabetes, dyslipidemia, and immediate poor fetal outcomes [42]. There is therefore a need to monitor for and manage pregnancy-related hypertensive disorders. Our study suggests several clinical proxies for anemia in pregnancy: low BMI, weight, and waist and hip circumferences. Moreover, elevated blood pressure may be a clinical harbinger for elevated maternal hemoglobin levels. The diagnostic and predictive utility of these clinical signs in anemia in pregnancy need to be further evaluated.

From this study, the prevalence of anemia in pregnancy was lower than in similar studies done in Kisugu-Kampala (25.8%) [19] and Gulu (22.1%) [11]. This finding is despite the fact that the majority of women enrolled in this study was in the second or third trimester of pregnancy where there is maximal hemodilution and demand for hematopoietic nutrients. This could be explained by the fact that the majority of the mothers enrolled in this study had a high level of education and thus were in a position to comprehend and heed to general public health measures to prevent anemia in pregnancy like adequate nutrition and prophylaxis with iron tablets during pregnancy.

In addition, the majority of mothers in our study were pregnant for at least the second time and thus could already have received information on the prevention of anemia in pregnancy during their index pregnancy, which they could have followed even before for their first antenatal care visit during the subsequent pregnancy. The majority of the pregnant mothers in this study were also employed and thus were more likely to have a better socioeconomic status than those unemployed and hence were able to afford nutritious meals during their pregnancy.

In this cross-sectional study, no risk factors were associated with anemia in pregnancy as shown by a lack of a statistically significant association between any of the variables and anemia in our study population. This is in contrast with other similar studies done in Uganda that identified lower gestational age, prima gravidity, unemployment of the pregnant mother [19], being a housewife, and lower level of education attained by the mother [11] as risk factors for anemia in pregnancy. Since our study had mostly multigravida mothers at higher gestational ages with most of them being employed and with at least a secondary level education qualification, this could explain the lack of any identifiable risk or associated factors with anemia in pregnancy from our study.

Our study is not without limitations. Firstly, in the cross-sectional study, we did not perform examination of the peripheral blood films, iron studies, and other tests for micronutrient deficiencies to characterize the anemia. However, we measured the MCV which has a modest sensitivity and specificity for iron deficiency in pregnancy [43, 44]. We also did not evaluate some common causes of anemia in Uganda such as helminth and malaria infections. While establishing the causes of anemia in this population as not the primary objective of the study, we evaluated some risk factors for anemia such as HIV infection, rural residence, and nutritional status (using anthropometric measurements as a proxy). Additionally, we could not exclude anemia preceding pregnancy among these women. However, we observed anemia in the second and third trimester only, with women in the third trimester posting a higher prevalence. This suggests that pregnancy preceded anemia and anemia progressed in severity later in pregnancy. This, however, needs to be further evaluated with a prospective study. Furthermore, our data does not include important anthropometric measurements such as the mid upper arm circumference. Nevertheless, the BMI, used in our study, has a very strong correlation with the MUAC among pregnant women attending antenatal care [45]. The heterogeneity of our meta-analysis must be taken into consideration while interpreting the results of this study. There was a mixture of community and antenatal care-based studies recruiting both the general population of pregnant women but also those in some specific trimesters. However, results from a less heterogeneous group in a sensitivity analysis were consistent with the overall results.

The strength of our study is in its design, combining data from a cross-sectional study and pooling it with studies from across the country with a total population of over 14,000 pregnant women. Thus, the findings from this study may reflect the true burden of anemia in pregnancy in the country.

## Conclusions

Anemia complicates nearly one third of pregnancies in Uganda, making it of a moderate public health significance. Despite our cross-sectional study having a lower prevalence compared to other studies in Uganda, these findings further confirm that anemia in pregnancy is still of public health significance and is likely to have nutritional causes, requiring targeted interventions. A larger study would be necessary to demonstrate potential use of basic clinical parameters such as weight or blood pressure as screening predictors for anemia in pregnancy.

## Supplementary Information

**Additional file 1:.** Data collection tool.

**Additional file 2:.** PRISMA checklist.

**Additional file 3:.** Search Strategy.

**Additional file 4:.** Risk of Bias Assessment of Individual Studies using Modified Newcastle Ottawa Scale.

## Data Availability

The datasets used and/or analyzed during the current study are available from the corresponding author on reasonable request.

## Abbreviations

MCV: Mean corpuscular volume
UDHS: Uganda Demographic and Health Survey
WHO: World Health Organization
Hb: Hemoglobin
