# Supplementary material for: Anemia in Ugandan pregnant women: a cross-sectional, systematic review and meta-analysis study
Source: Trop Med Health. 2021 Mar 1;49:19. doi: 10.1186/s41182-021-00309-z (PMC7919073; doi:10.1186/s41182-021-00309-z)
Supplement: Supplementary file 1 — Additional file 1:. Data collection tool. [file 41182_2021_309_MOESM1_ESM.docx]

## DATA COLLECTION TOOL

1. **PARTICIPANT’S IDENTIFIERS**

Participant’s Initials: |___|___|___| Study ID: |F|B|___|___|__|__|

Date of enrolment: |___|___|/|___|___|/|___|___|___|___| (DD/MM/YYYY)

Antenatal care visit number: ________________________________________

1. **SOCIO-DEMOGRAPHIC VARIABLES**
2. Age, **|___|___|** years
3. Marital status

1. Married [ ] 2. Single [ ] 3. Widowed [ ]

1. Level of Education

1. None [ ] 2. Primary [ ] 3. Secondary [ ] 4. Tertiary [ ]

1. Occupation Group

1. Unemployed [ ] 2. Unskilled Worker [ ]

3. Skilled Worker [ ] 4. Business [ ]

5. Professional [ ], Profession ________________

1. Smoking status

1. Never [ ] 2. Current (< 6 months) [ ] 3. Former (>6 months) [ ]

1. Alcohol usage

1. Never [ ] 2. Current (< 6 months) [ ] 3. Former (>6 months) [ ]

1. Family History of Diabetes

1. Yes [ ] 2. No [ ]

1. Area of Residence

District. ……………………………

1. Rural [ ] 2. Peri-urban [ ]

1. **HIV VARIABLES**
2. HIV status

If known HIV-positive

Duration of disease since diagnosis: __________

Current CD4:__________ Current VL:__________ ART regimen: ____________

If HIV status unknown, Routine Counselling and HIV Testing

1. Positive [ ] 2. Negative [ ]

1. **FAMILY SIZE**
2. Crowding index

1. 1-4 Persons/House [ ], n= _________

2. ≥5 Persons/House [ ], n= __________

1. **OBSTETRIC VARIABLES**
2. Gravidity: [__________]
3. Parity: [__________]
4. Last Normal Menstrual Period: [__________]
5. **ANTHROPOMETRY**
6. Weight: [__________]/Kilogram
7. Height: [__________]/Metre
8. Waist circumference: [__________]/Centimetre
9. Hip circumference [__________]/Centimetre
10. Blood pressure 1 [_____/_____] 2 [_____/_____]
11. **LABORATORY VARIABLES**
12. Haemoglobin level
13. Mean corpuscular volume
