# Supplementary material for: Anemia in Ugandan pregnant women: a cross-sectional, systematic review and meta-analysis study
Source: Trop Med Health. 2021 Mar 1;49:19. doi: 10.1186/s41182-021-00309-z (PMC7919073; doi:10.1186/s41182-021-00309-z)
Supplement: Supplementary file 3 — Additional file 3:. Search Strategy. [file 41182_2021_309_MOESM3_ESM.docx]

**Anemia in Ugandan pregnant women: a cross-sectional study, systematic review and meta-analysis**

**Table 1. Search strategy**

| **Database** | **Search strings** |
| --- | --- |
| EMBASE, Medline, Cochrane, ICTRP, and ClinicalTrials.gov. | “burden” OR “prevalence” OR “incidence” AND “anaemia” OR “anemia” OR “low haemoglobin” OR “haemoglobin <11 g/dl” AND “pregnancy” OR “pregnant” OR “gestation” AND "Uganda" |
| African Journals Online (AJOL) | “anaemia in pregnancy in Uganda” OR “anemia in pregnancy in Uganda” |
