# Supplementary material for: Anemia in Ugandan pregnant women: a cross-sectional, systematic review and meta-analysis study
Source: Trop Med Health. 2021 Mar 1;49:19. doi: 10.1186/s41182-021-00309-z (PMC7919073; doi:10.1186/s41182-021-00309-z)
Supplement: Supplementary file 4 — Additional file 4:. Risk of Bias Assessment of Individual Studies using Modified Newcastle Ottawa Scale. [file 41182_2021_309_MOESM4_ESM.docx]

**Anemia in Ugandan pregnant women: a cross-sectional study, systematic review and meta-analysis**

Supplementary File 1. Risk of Bias Assessment of Individual Studies using Modified Newcastle Ottawa Scale.

Adopted from: <https://journals.plos.org/plosone/article/file?type=supplementary&id=info:doi/10.1371/journal.pone.0147601.s001>

| **Study** | **Represent-ativeness *** | **Sample size *** | **Non-respondents *** | **Ascertainment of exposure **** | **Compara-bility **** | **Assessment of outcome **** | **Statistical test *** | **Total** | **Quality** |
| --- | --- | --- | --- | --- | --- | --- | --- | --- | --- |
| Bongomin et al (Present Study) | * | * | * | ** |  | ** | * | ******** | Very good |
| Kasumba et al (2000) | * |  |  | ** | * | ** | * | ******* | Good |
| Okia et al (2019) | * | * | * | ** |  | ** | * | ******** | Very good |
| Ndyomugenyi et al (2008) | * | * | * | ** | ** | ** | * | ********** | Excellent |
| Obai et al (2016) | * | * | * | ** |  | ** | * | ******** | Very good |
| Namusoke et al (2010) | * |  |  | ** | * | ** | * | ******* | Good |
| Mahamoud et al (2020) | * | * |  | ** |  | ** | * | ******* | Good |
| Braun et al (2015) | * | * | * | ** | * | ** | * | ********* | Very good |
| Nekaka et al (2020) | * | * | * | ** | * | ** | * | ********* | Very good |
| Baingana et al (2014) | * |  |  | ** | ** | ** | * | ******** | Very good |
| Finkelstein et al (2020) |  | * |  | ** | * | ** | * | ******* | Good |
| Ononge et al (2014) | * | * |  | ** | ** | ** | * | ********* | Very good |
| Ndibazza et al (2010) | * | * | * | ** | ** | ** | * | ********** | Excellent |
| Muhangi et al (2007) | * | * |  | ** | ** | ** | * | ********* | Very good |
| Arinaitwe et al (2013) | * | * | * | ** | * | ** | * | ********* | Very good |
| Mbonye et al (2008) | * | * | * | ** | * | ** | * | ********* | Very good |
| Mbule et al (2013) | * | * | * | ** |  | ** | * | ******** | Very good |
